# Gesture-controlled interactive three dimensional anatomy: a novel teaching tool in head and neck surgery

**DOI:** 10.1186/s40463-014-0038-2

**Published:** 2014-10-07

**Authors:** Jordan B Hochman, Bertram Unger, Jay Kraut, Justyn Pisa, Sabine Hombach-Klonisch

**Affiliations:** Neurotologic Surgery, Department of Otolaryngology - Head and Neck Surgery, Faculty of Medicine, University of Manitoba, GB421, 820 Sherbrook Street, Winnipeg, Manitoba Canada; Clinical Learning and Simulation Facility, Department of Medical Education, Faculty of Medicine, University of Manitoba, Winnipeg, Manitoba Canada; Department of Medical Education, Faculty of Medicine, University of Manitoba, Winnipeg, Manitoba Canada; Department of Otolaryngology - Head and Neck Surgery, Health Sciences Centre, Surgical Hearing Implant Program, GB421, 820 Sherbrook Street, Winnipeg, Manitoba Canada; Department of Human Anatomy and Cell Science, Faculty of Medicine, University of Manitoba, Winnipeg, Manitoba Canada

**Keywords:** Interactive, 3D model, Gesture controlled, Virtual reality, Haptic, Temporal bone

## Abstract

**Background:**

There is a need for innovative anatomic teaching tools. This paper describes a three dimensional (3D) tool employing the Microsoft Kinect™. Using this instrument, 3D temporal bone anatomy can be manipulated with the use of hand gestures, in the absence of mouse or keyboard.

**Methods:**

CT Temporal bone data is imported into an image processing program and segmented. This information is then exported in polygonal mesh format to an in-house designed 3D graphics engine with an integrated Microsoft Kinect™. Motion in the virtual environment is controlled by tracking hand position relative to the user’s left shoulder.

**Results:**

The tool successfully tracked scene depth and user joint locations. This permitted gesture-based control over the entire 3D environment. Stereoscopy was deemed appropriate with significant object projection, while still maintaining the operator’s ability to resolve image details. Specific anatomical structures can be selected from within the larger virtual environment. These structures can be extracted and rotated at the discretion of the user. Voice command employing the Kinect’s™ intrinsic speech library was also implemented, but is easily confounded by environmental noise.

**Conclusion:**

There is a need for the development of virtual anatomy models to complement traditional education. Initial development is time intensive. Nonetheless, our novel gesture-controlled interactive 3D model of the temporal bone represents a promising interactive teaching tool utilizing a novel interface.

## Introduction

Three-dimensional (3D) virtual imagery can be an important tool for understanding the spatial relationships between distinct anatomical structures. This is particularly relevant in regions for which the classical dissection technique has limitations. For example, the complexity and microscopic nature of head and neck anatomy has proven to be an ongoing challenge for learners [1]. Within the temporal bone, there are considerable soft tissue structures, densely situated in bone, making severe demands on visuo-spatial capabilities. New learners and Senior residents must grapple with complex normative and pathologic conditions, some which occur only infrequently. Here, novel tools are needed to facilitate spatial anatomic learning and to adequately prepare the professional trainee for the practical demands of surgery. Previous research has indicated that the learning experience of students is positively affected when 3D teaching tools are used in parallel with traditional teaching methods [2]. 3D computer simulations have been introduced in the teaching of the middle and inner ear [3], the orbital anatomy [4], and dental anatomy [5], with encouraging results.

Medical students still learn the anatomy of this region primarily through illustrated texts, many of which have been in print for decades [6-8], but the dissection of the temporal bone itself is usually limited to senior trainees, largely due to the relative scarcity of available samples for practicing operative approaches.

With the advent of high-speed computing, 3D graphical models of complex anatomy have become possible [3,9-14]. Actual interaction with 3D anatomical models can occur at several levels. In the simplest form they may involve allowing the user to examine an object in 3D or from different viewpoints [9,15-18]. In more complex cases, a user may be able to select components for closer study, move them about and examine supplementary data such as labels, radiographs and animations [2,3,19-27]. At the highest levels, users may interact in a natural way with the model, moving it by grasping it with a hand or altering it by cutting or drilling with a tool [10,28]. The addition of gesture-based interaction to stereoscopic models combines intuitive interaction with immersive visualization. It is postulated that such a system could alleviate cognitive overload by providing a learner with an environment in which their natural actions act on objects, without the need for complex input devices.

While the technology and accompanying literature surrounding 3D imagery develops, education needs to continue to advance in the setting of both time and fiscal constraints. In this paper we describe a novel gesture-controlled 3D teaching tool in which the three dimensional temporal bone anatomy is manipulated with the use of hand gestures through a Microsoft Kinect™, in the absence of mouse and keyboard. Key structures are easily maneuvered and can be removed and better examined in reference to the whole. This novel tool provides a learning environment in which the physical involvement of the user may enhance the learning experience and increase motivation.

## Methods

In order to take advantage of recent advances in technology we have developed a 3D stereoscopic display which uses the Microsoft Kinect™ (Microsoft Corporation, Redmond, Washington, USA) to allow gesture control of anatomical images. Images can be selected, translated, magnified and rotated with simple body motions. The system uses 3D models extracted from CT data by segmentation of anatomical structures of interest. The models are then displayed stereoscopically by a 3D graphics engine which incorporates gesture control from the Microsoft Kinect™. What follows is a description of the system and the process by which anatomical information is converted from tomographic data to a gesture-based anatomy teaching tool.

Our aim is to provide a teaching tool for patient-specific anatomy. To facilitate this, we use actual CT images as the basis. In our prototype, 0.15 mm slice thickness cadaveric temporal bone images (General Electric MicroCT - eXplore speCZT, 0.150 mm thickness) are acquired and imported to a 3D image processing program (Mimics v. 11.02, Materialise NV, Leuven, Belgium). The dataset is resampled to a slice interval of 0.1 mm to help volume interpolation. Anatomical regions of interest, such as the temporal bone, internal carotid artery and facial nerve are identified by segmentation. Initial segmentation is carried out by thresholding CT data by density. For example, the temporal bone is identified by retaining all voxels with densities between 382 and 3071 Hounsfield units (HU). Soft tissue regions and ossicles are manually segmented by visual inspection of the data while varying the density threshold; an expert then inspects the margins of the rough segmentation and adds or removes voxels as needed, based on knowledge of the anatomy. For example, with the contrast set to HU less than -50, the tympanic membrane can be partly resolved and the margins of the membrane extrapolated by estimation. To ensure that the membrane will appear intact in the final model, it is thickened to 2-3 voxels.

**Figure 1:**
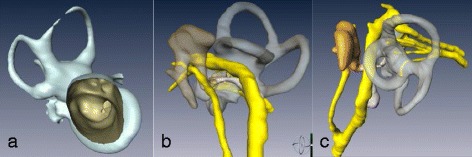
**Segmented 3D temporal bone anatomy. a)** Cochleo-vestibular apparatus with medial to lateral orientation and direct view into the internal auditory canal. **b)** Sagittal view of external meatus. Note the ossicular network (brown), vertical segment of the facial nerve (yellow), and cochleo-vestibular apparatus (transparent grey). **c)** View perpendicular to the internal acoustic meatus with appreciation of facial, cochlear and both inferior and superior vestibular nerves (yellow).

The segmented anatomical models are converted to 3D polygonal mesh format and exported in stereolithography file format (STL) (Figure 1). The resulting models can be displayed in 3D, using a commercially available 3D graphics card (Nvidia GeForce GTX560 - Santa Clara, California, USA), active shutter glasses and either a 3D capable monitor or projector. We have developed our own 3D anatomical graphics engine which loads and renders multiple large polygonal mesh models in 3D and allows users to manipulate camera positions as well as select and manipulate individual models.

Our graphics engine is developed in Microsoft Visual Studios 2008 using the Microsoft Foundation Class software library and the C++ programming language. The Microsoft Kinect™ Software Development Kit (MKSDK) and the NVidia Application Programming Interface (API) were integrated. To render in 3D with stereoscopy (Nvidia’s 3D vision) the DirectX 11.0 API is employed. 3D vision is automatically engaged when an application is set to full screen. The hardware and software requirements needed to run our engine are widely available and accessible to the general user.

**Figure 2:**
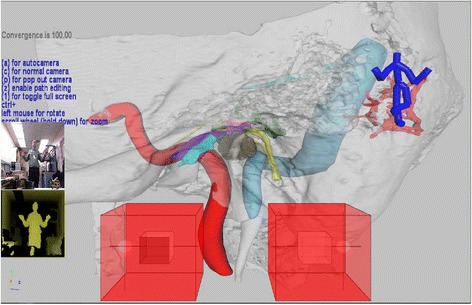
**Screen shot of 3D Kinect™** **gesture controlled demo.** The large red cubes in the forefront govern navigation with the left hand controlling translational movement, and the right hand controlling rotation and orientation. The smaller white cubes, set inside the control cubes, are used to visualize hand locations. The user is represented pictorially by colour camera and infrared depth sensor on the left and graphically by the avatar in the top right.

**Figure 3:**
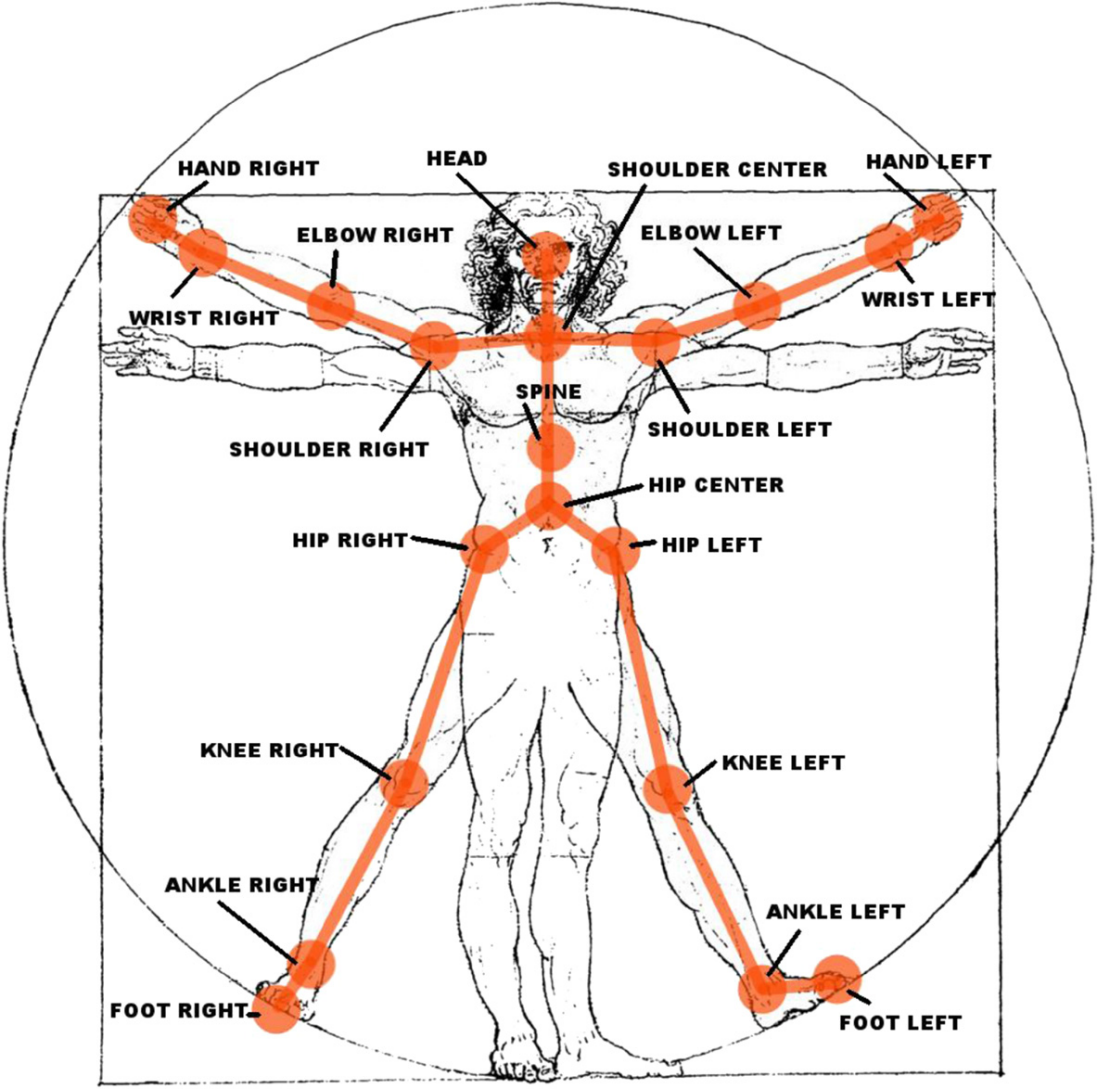
**Joints identified and tracked by the Kinect™.** An in-house generated image depicting the use of the joints by the Kinect for gesture control. No copyright should be required (2^nd^ Item from Editorial staff).

The MKSDK uses input from a colour camera and infrared depth sensor to detect human motion. It provides information on scene depth and color (Figure 2) based on the joint locations (Figure 3). It also contains an intrinsic speech library that facilitates speech recognition using a built-in microphone. Using the MKSDK, the software is able to integrate user body motions detected by the Kinect™ into our anatomical graphics engine.

## Results

**Figure 4:**
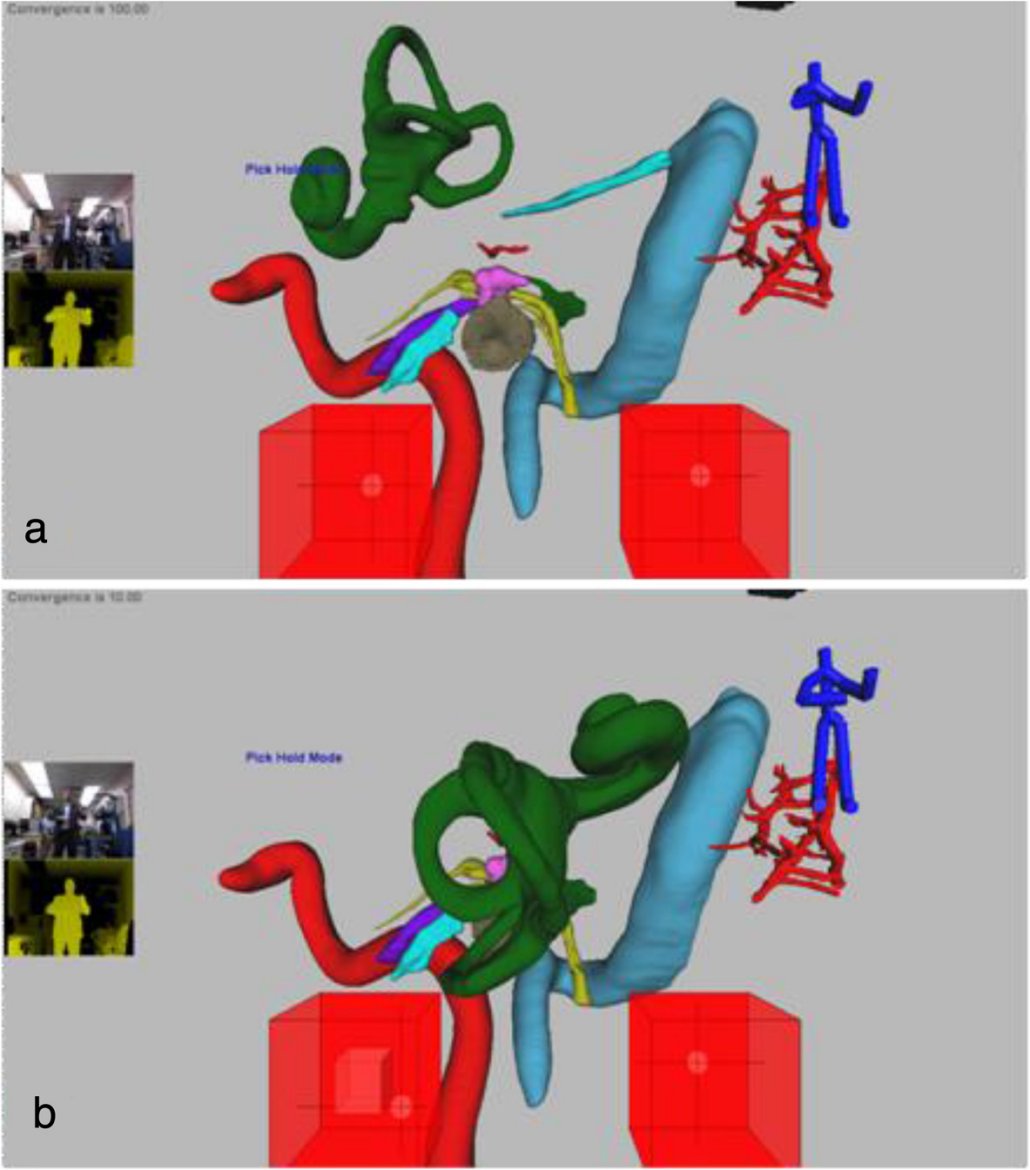
**3D anatomy tool selection mode with cochleo-vestibular apparatus brought to forefront.** Objects may be manipulated both by gesture and voice control. **a)** Cochleo-vestibular apparatus, having been selected, in transit towards viewer. **b)** Cochleo-vestibular apparatus “popped” out of screen in 3D and rotated by 180°. It may be translated, magnified or rotated under user control using gestures. The users are first author Jordan Hochman and 2^nd^ author Bert Unger.

Our software uses the Kinect™ to allow an operator to navigate in 3D space and to select specific anatomical structures of interest from within the larger virtual environment (Figure 4). These structures can then be extracted and rotated in all planes at the discretion of the user.

To move in 3D space, both the left and right hand are tracked relative to the position of the left shoulder. The left hand controls translational movement, and the right hand controls rotation and orientation. Two cubes, shown at the bottom of both Figures 2 and 4, are used to visualize hand locations. A preset distance from the hand to the shoulder is defined as the center of each cube. When the hand, represented by a small sphere, is centered in a cube, no movement or rotation occurs. As the hand moves away from the center, camera movement or rotation is proportional to the hand’s distance from the center. When the user’s hand lies outside of the cube for several seconds, motion control of the scene is disabled. Motion control can be re-enabled by again placing one’s hand in the center reference position.

The NVidia API allows the software to control depth and convergence of 3D vision in our system. Depth settings control the illusion of depth in the 3D image; convergence settings control the distance from the camera and at which objects appear to “pop” out of the screen. If these settings are too low then 3D stereoscopy may not be noticeable, however if too large, there can be divergence and the stereoscopy may not be resolved as a single image, resulting in eye-strain.

When the camera is at a desired location, the user can switch modes to select objects of interest for closer inspection. The operator switches modes by either tapping their left shoulder with their right hand, or employing an audio command. When the selection mode is activated, the left cube controls a sphere that can move within the 3D scene to highlight any desired structure. Once an object is highlighted it can then be selected by another shoulder tap or an audio command. Once an object is selected (Figure 4), the left hand controls the location of the structure while the right hand controls its orientation. The 3D vision effect is set to bring the selected object, towards the user, enabling a “pop out” so the anatomy can be observed more closely and manipulated separately from the larger model.

## Discussion

New technologies are advocated, not to replace but rather, to complement classic learning. These modalities are best perceived as fueling a renaissance in anatomy learning as opposed to supplanting cadaveric education. They represent a promising opportunity in medical education. Successful integration into standard training and patient care requires a significant interplay between anatomists, clinicians and engineering. Collaborative development of educational and manipulative tools needs to advance before global acceptance is assured.

Requisite to any teaching model is the recognition that anatomy is fundamental for responsible and effective medical education and patient management and the deconstruction of anatomic education and the associated undermining of crucial knowledge and skills may lead to under-qualified doctors. Medical education needs to be enduring and not solely pertinent to exam purposes. Patient oriented and safe care includes a sound anatomical basis provided during formative years in association with lifelong regular learning.

Initial costs in setup and design of 3D digital medical education tools may seem prohibitive. A cost comparison between physical and digital dissection was undertaken by Hisley *et al*. in 2007 [19]. Physical dissection appeared more economical when a singular cadaver was compared to initial setup of a virtual dissected specimen. However, even accounting for multiple work stations and the accrual of a broad anatomic library, digital dissection quickly becomes a less expensive option when considered longitudinally.

Unfortunately the development of three dimensional models is time intensive. The constructed images are highly accurate and drawn from real anatomy but ultimately remain a stylized abstraction. Additionally, it is difficult to determine the appropriate level of detail to include, as a teaching module may be used by disparate learners. Dissimilar file formats are employed by different institutions and the sharing of information/crafted modules are complicated for proprietary programs [29]. If the data is obtained from histologic samples, difficulties inherent in embalming, freezing and slicing may cause irregularities within the data sets and ultimate inaccuracies in the anatomy.

Case-specific three dimensional visualization is now possible. The process is limited by the requisite time for segmentation. However, complex, variant and unusual cases may dictate such an investment. The near future holds the promise of automated segmentation [30,31], further encouraging these newer technologies. The current iteration of the Kinect™ can also be employed in the operative theatre allowing the user to maintain sterility while providing valuable spatial information on the relationship between normal and pathologic anatomical structures, with an aim of preserving the former.

## Conclusion

There is a great need for the development of advanced virtual anatomy models to complement traditional education. Our novel gesture-controlled interactive 3D model of temporal bone anatomy comprises a promising teaching tool, not only for the early learner, but in particular for the advanced learner with an aim to better prepare professionals for advanced spatial comprehension in surgical practice.
